# Virtual non-contrast images calculated from dual-energy CT shoulder arthrography improve the detection of intraarticular loose bodies

**DOI:** 10.1007/s00256-022-04007-7

**Published:** 2022-02-11

**Authors:** Christoph Stern, Dimitri N. Graf, Samy Bouaicha, Karl Wieser, Andrea B. Rosskopf, Reto Sutter

**Affiliations:** 1 Radiology, Balgrist University Hospital, Forchstrasse 340, 8008 Zurich, Switzerland; 2 Faculty of Medicine, University of Zurich, Zurich, Switzerland; 3 Department of Orthopaedic Surgery, Balgrist University Hospital, Forchstrasse 340, 8008 Zurich, Switzerland

**Keywords:** Computed tomography, X-ray, Arthrography, Shoulder, Image enhancement, Osteochondral body

## Abstract

**Objective:**

This study aims to evaluate the image quality of virtual non-contrast (VNC) images calculated from dual-energy CT shoulder arthrography (DECT-A) and their ability to detect periosteal calcifications and intraarticular loose bodies.

**Materials and methods:**

In 129 shoulders of 123 patients, DECT arthrography (80 kV/140 kV) was performed with diluted iodinated contrast material (80 mg/ml). VNC images were calculated with image postprocessing. VNC image quality (1 = worst, 5 = best), dose parameters, and CT numbers (intraarticular iodine, muscle, VNC joint fluid density) were assessed. Image contrast (iodine/muscle) and percentage of iodine removal were calculated. Two independent readers evaluated VNC and DECT-A images for periosteal calcifications and intraarticular loose bodies, and diagnostic confidence (1 = low, 4 = very high) was assessed.

**Results:**

VNC images (129/129) were of good quality (median 4 (3–4)), and the mean effective dose of DECT-A scans was 2.21 mSv (± 1.0 mSv). CT numbers of iodine, muscle, and VNC joint fluid density were mean 1017.6 HU (± 251.6 HU), 64.6 HU (± 8.2 HU), and 85.3 HU (± 39.5 HU), respectively. Image contrast was mean 953.1 HU (± 251 HU) on DECT-A and 31.3 HU (± 32.3 HU) on VNC images. Iodine removal on VNC images was 91% on average. No difference was observed in the detection of periosteal calcifications between VNC (*n* = 25) and DECT-A images (*n* = 21) (*p* = 0.29), while the detection of intraarticular loose bodies was superior on VNC images (14 vs. 7; *p* = 0.02). Diagnostic confidence was higher on VNC images for both periosteal calcifications (median 3 (3–3) vs. 3 (3–3); *p* = 0.009) and intraarticular loose bodies (median 3 (3–4) vs. 3 (3–3); *p* < 0.001).

**Conclusion:**

VNC images from DECT shoulder arthrography are superior to DECT-A images for the detection of intraarticular loose bodies and increase the confidence in detecting periosteal calcifications.

**Supplementary Information:**

The online version contains supplementary material available at 10.1007/s00256-022-04007-7.

## Introduction

Osseous fragments or intraarticular loose bodies in patients with previous shoulder dislocation or osteoarthritis can cause symptoms such as locking, snapping, pain, and reduced range of motion or instability [1–3]. In such patients, CT of the shoulder is routinely performed to localize and quantify osseous fragments or intraarticular loose bodies. Furthermore, CT allows to evaluate the morphology of the humeral head and of the glenoid and to assess the amount of humeral and/or glenoid bone loss. In a subset of patients, arthrography is performed before the CT scan, which allows for the evaluation of the cartilage, labrum, and the rotator cuff. However, on cross-sectional images after arthrography, thin periosteal calcifications or intraarticular loose bodies might be obscured by the dense intraarticular iodinated contrast material, whereas otherwise they are well visible on unenhanced CT.

Dual-energy CT (DECT) with its ability to characterize tissues and other material (e.g., iodine) because of different attenuation values at different energy levels allows for the detection of gout [4] and bone marrow edema [5] and reduces artifacts around metal implants [6]. Furthermore, with DECT, both blended CT arthrography and virtual non-contrast (VNC) images can be acquired in a single scan without additional radiation dose to the patient [7]. VNC images are calculated by subtraction of iodine [8, 9] and have been successfully used in liver [10, 11], renal [12], and vascular imaging [13], making true unenhanced images obsolete for a comprehensive evaluation. Moreover, VNC images from dual-energy CT shoulder arthrography (DECT-A) with iodine removal allow the calculation of accurate 3D reformats of the glenoid for assessment of bone loss [7].

To our knowledge, VNC images have not been evaluated in shoulder imaging for their ability to detect periosteal calcifications or intraarticular loose bodies. Only one reported case exists that showed the visibility of a scapular fracture fragment on a DECT-A, virtual monoenergetic and virtual non-contrast image [14]. We set out to generate and evaluate VNC images in shoulder patients who received dual-energy CT after arthrography as a clinical routine examination. Our hypothesis was that VNC images can successfully differentiate periosteal calcification from contrast material due to a labral or periosteal tear and improve the detectability of intraarticular loose bodies.

Therefore, the purpose of this study was to evaluate the quality of VNC images and to compare the detectability of periosteal calcification and intraarticular loose bodies on VNC and on DECT-A images.

## Materials and methods

This retrospective study, which was performed at a single center, was approved by the cantonal ethics committee and was conducted according to the Declaration of Helsinki.

### Study population

The picture archiving and communication system (PACS) of Balgrist University Hospital was queried to identify patients who received a clinical dual-energy CT scan of the shoulder after arthrography because of dysplasia, glenohumeral instability, history of shoulder dislocation or humeral head trauma, pain, rotator cuff tear, osteoarthritis, or for preoperative assessment between July 2019 and February 2021. Males and females with an age of 18 years or older were included. Exclusion criteria were metal screws or metal anchors in the glenoid after surgery, incorrect dosage of intraarticular contrast material at arthrography, and extraarticular contrast injection. A third of the study population have been reported in a previous study with a different focus [7].

### Arthrography and dual-energy CT technique

Arthrography with a total volume of 12 ml was performed in all patients before the CT scans. At Balgrist University Hospital, an anterior approach through the rotator cuff interval is routinely used to inject the diluted iodinated contrast material into the glenohumeral joint under conventional fluoroscopy [15]. A solution of 80 mg iodine per milliliter was used for all injections which was achieved by injecting 1 ml of local anesthetics followed by 11 ml of diluted contrast material. For the dilution, 7 ml Iopamiro 200 (Iopamidol) was mixed with 9 ml NaCl 0.9% (total 16 ml) from which 11 ml was used.

Within 15 min after arthrography, all patients received a dual-energy CT scan of the shoulder at Balgrist University Hospital either on a 64-slice CT scanner (SOMATOM Definition AS, Siemens Healthineers, Erlangen, Germany) or on a 128-slice CT scanner (SOMATOM Edge Plus, Siemens Healthineers, Erlangen, Germany). As no specific manufacturer protocol was available, the protocol for the liver VNC application was used with adaptions for the shoulder: all examinations were performed in sequential technique (1st scan 80 kV, 2nd scan 140 kV; same coverage in *z*-axis), with automated tube current modulation (CARE Dose4D, reference 240 mAs for 80 kV and 57 mAs for 140 kV), with a collimation width of 0.6 mm, a rotation time of 0.5 s and a pitch of 0.8.

The CT machine automatically splits the total dose between the 80 and 140 kV scan. Settings were adjusted to the dose parameters of a single-energy scan of the shoulder at 120 kV (reference 150 mAs).

### Image reconstruction and postprocessing

Axial images (0.75 mm) in the bone (Br 57) and soft tissue kernel (Qr 40) were reconstructed for both the 80 kV and 140 kV scan. Furthermore, the 80 kV and 140 kV scans were used for calculation of blended axial CT arthrography (DECT-A) images (0.75 mm) in bone kernel (Br 57), applying a mixing ratio of 0.3:0.7.

**Fig. 1:**
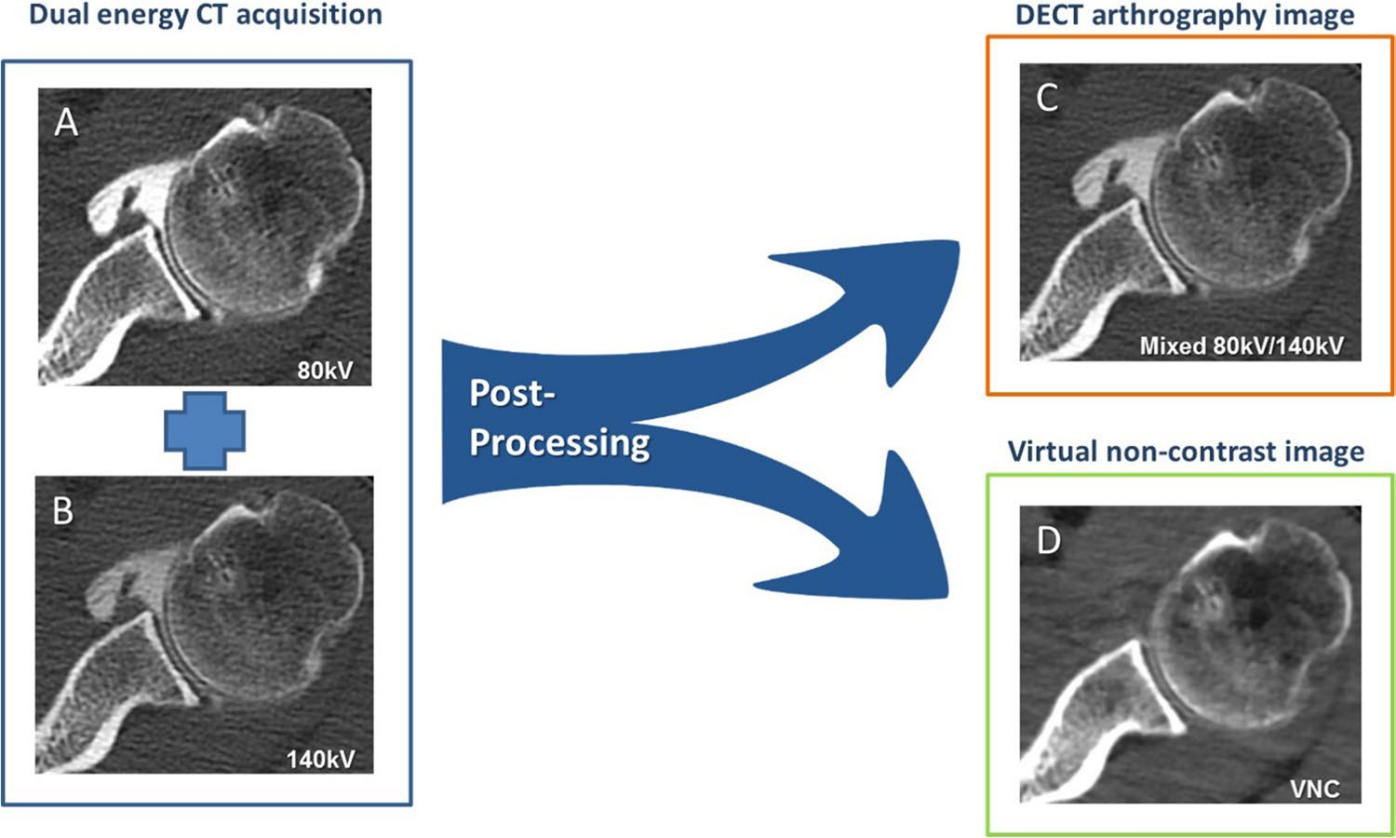
Image acquisition and postprocessing of dual-energy CT shoulder arthrography. 2 datasets, one with 80 kV (**A**) and one with 140 kV tube voltage (**B**), are acquired in the same shoulder with the dual-energy CT scan. DECT arthrography images (80 kV/140 kV) (**C**) with a mixing ratio of 0.3:0.7 and virtual non-contrast images (**D**) are calculated with image postprocessing from (**A**) and (**B**)

Virtual non-contrast images with a 0.75-mm section thickness were calculated in syngo.via (VB 30, Siemens Healthineers, Erlangen, Germany) using the axial 80 kV and 140 kV dataset (0.75 mm) in soft tissue kernel (Qr 40). Images were loaded into the dual-energy viewer of syngo.via, and the shoulder VNC application was used for image calculation, which was adapted from the liver VNC application to display higher Hounsfield units (Fig. 1).

DECT-A images were displayed with a window width of 2500 HU and a window level of 600 HU, while for VNC images, the window width and level was 600 HU and 150 HU, respectively. Readers were free to adjust the windowing according to their own preference if necessary.

### Image analysis

Two musculoskeletal radiologists (C.S. (reader 1) and D.G. (reader 2), both with 8 years of experience), interpreted images independently on a PACS workstation. Images were anonymized, and both readers were blinded to each other and were blinded to clinical information and imaging results.

#### Quantitative image analysis

The dose report of every examination was available and was used to extract the scan length and CT dose parameters: tube current–time product (mAs), volume CT dose index (CTDIvol), and dose length product (DLP). The effective dose was estimated with the DLP which was multiplied with a standard conversion factor *k* for the adult chest of 0.014 mSv/mGy* [16].

Reader 1 measured CT values (HU) of the intraarticular iodinated contrast material (iodine DECT-A) and of the deltoid muscle on axial DECT arthrography images using regions of interest (ROI) of equal size (20 mm^2^). Furthermore, a ROI (20 mm^2^) was placed in the VNC images to measure the CT values (HU) of joint fluid after iodine subtraction (iodine VNC). To ensure identical ROI positioning in both datasets, the copy and paste function of the PACS were used. Calculation of image contrast was performed for DECT arthrography and VNC images: iodine DECT-A (HU) — muscle (HU) and iodine VNC (HU) — muscle (HU), respectively. Furthermore, the percentage of iodine subtraction on VNC images was calculated: (iodine DECT-A – iodine VNC) / iodine DECT-A × 100.

#### Qualitative image analysis

On a 5-point Likert scale, both readers rated the overall image quality of the blended DECT-A images (1 = poor, 2 = fair, 3 = moderate, 4 = good, 5 = excellent) and of the virtual non-contrast images (1 = poor, 2 = fair, 3 = moderate, 4 = good, 5 = excellent). Supplementary Table 1 shows definition of ratings.

#### Imaging findings

Both readers evaluated the virtual non-contrast images and axial DECT arthrography images for the presence or absence of periosteal calcifications and intraarticular loose bodies. Diagnostic confidence for each finding was rated on a 4-point Likert scale (1 = low, 2 = moderate, 3 = high, 4 = very high). Reader 1 first interpreted VNC images and then DECT-A images, whereas reader 2 evaluated the images in the opposite order. The interval between image analysis was 2 months for both readers. Four months past the image reading, a consensus reading with an independent third reader (R.S. with 16 years of experience) was performed to confirm or decline imaging findings based on simultaneous interpretation of VNC and DECT-A images, additional examinations (CT, MRI, x-ray), and arthroscopic reports, if available.

### Statistical analysis

We used general descriptive statistics and reported the median with 25th percentile (Q1) and 75th percentile (Q3) and the mean with standard deviation (SD).

All imaging findings of reader 1 were shown in the *Results* section, and agreement between readers 1 and 2 was reported. For the virtual non-contrast and the DECT arthrography images, the prevalence of periosteal calcifications and intraarticular loose bodies was calculated and compared using the McNemar test. The Wilcoxon signed-rank test was used to compare diagnostic confidence of every image finding. Kappa statistics (ĸ*)* was calculated to measure interreader agreement with interpretation of effect size for ĸ as slight (0–0.20), fair (0.21–0.40), moderate (0.41–0.60), substantial (0.61–0.80), or excellent (0.81–1.00) [17].

SPSS (version 26, IBM Corporation, Armonk, NY) was used for statistical analysis and any value of *p* < 0.05 was considered statistically significant.

## Results

### Study participants

The search of the PACS revealed 186 patients who received a dual-energy CT of the shoulder after arthrography. Thirty-one patients declined informed consent and were excluded. Further exclusions were because of metal implants (8 patients), incorrect dosage of intraarticular contrast material at arthrography (5 patients), and complete or predominantly extraarticular injection of contrast material (19 patients). This resulted in a study group of 123 patients (95 male, 28 female; mean age 36.4 years ± 14.5 years [standard deviation]) and 129 shoulders (5 male patients and 1 female patient received DECT after arthrography of both shoulders).

#### CT parameters, effective dose, and quantitative image analysis

Table 1 shows the parameters of the DECT shoulder scans after arthrography. The estimated effective dose of the DECT-A scans was mean 2.21 mSv (± 1.0 mSv).

**Table 1:** CT scans: scan length and dose parameters

	Dual-energy CT shoulder after arthrography
Tube current–time product	**80 kV: reference 240 mAs** **140 kV: reference 57 mAs**
CTDI_vol_	**12.8 mGy** (± 5.6 mGy)
DLP	158.0 mGy*cm (± 73.8 mGy*cm)
Scan length	123 mm (± 12 mm)
Effective dose ^†^	**2.21 mSv** (± 1.0 mSv)

CT parameters were automatically adapted to patient size

^†^ effective dose (mSv) was estimated by multiplying the DLP with a standard conversion factor k for the adult thorax of 0.014 mSv/mGy*cm

Values are displayed as mean with standard deviation in parentheses

*Abbreviations: CTDIvol*, volume CT dose index; *DLP*, dose length product; *kV*, kilo volt; *mAs*, milliampere seconds; *mGy*, milligray; *mSv*, millisievert

Blended DECT arthrography images and virtual non-contrast images were successfully calculated for all shoulders (129/129, 100%).

The following CT values were measured: intraarticular iodinated contrast material with mean 1017.6 HU (± 251.6 HU) and deltoid muscle with mean 64.6 HU (± 8.2 HU) on DECT-A images and joint fluid density after iodine subtraction with mean 85.3 HU (± 39.5 HU) on VNC images. Image contrast on DECT-A images (iodine DECT-A (HU), muscle (HU)) was mean 953.1 HU (± 251 HU), while image contrast on VNC images (iodine VNC (HU), muscle (HU)) was mean 31.3 HU (± 32.3 HU). The percentage of intraarticular iodine subtraction on VNC images was 91% (± 4%) on average.

#### Qualitative image analysis

Both readers rated the quality of DECT arthrography images and virtual non-contrast images as good: reader 1 and 2 both rated DECT-A images median 4 (3–4) and VNC images median 4 (3–4).

#### Imaging findings

**Fig. 2:**
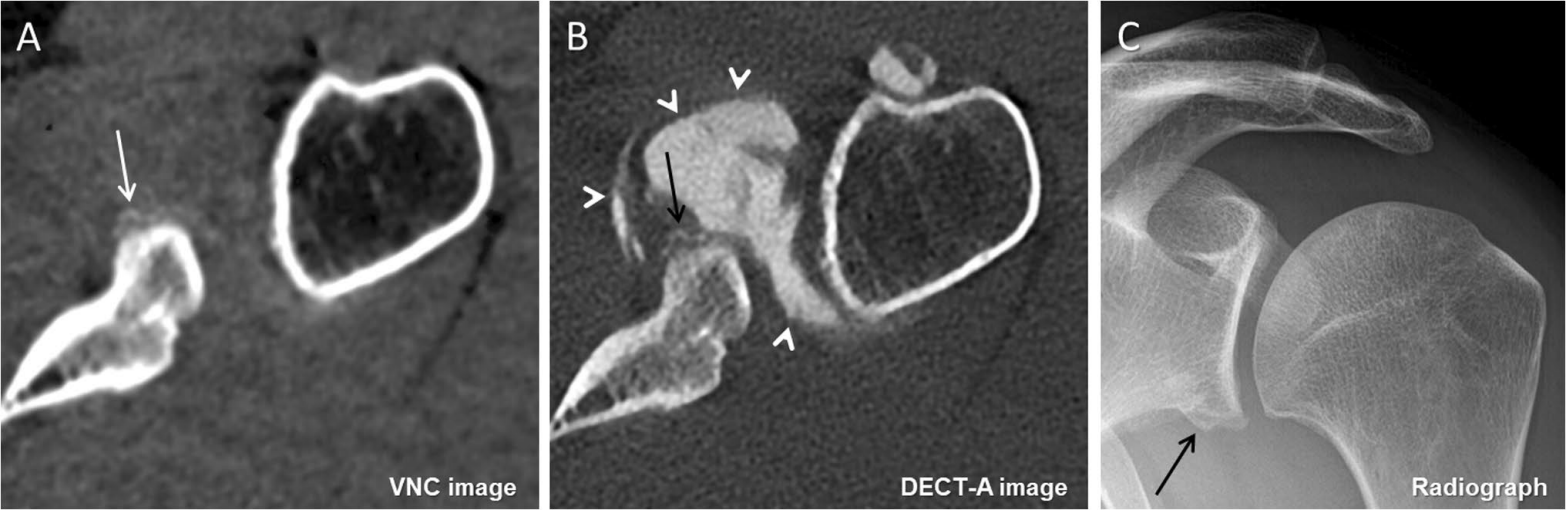
A 31-year-old male with recurrent anteroinferior shoulder dislocation. On the axial virtual non-contrast image (**A**), a thin periosteal calcification is visible (white arrow), which had been wrongly interpreted as labral-periosteal tear (black arrow) outlined by iodinated contrast material (arrowheads) on the axial DECT arthrography image (**B**). The coronal radiograph (**C**) confirms the shell-like periosteal calcification at the anteroinferior glenoid (arrow). The confidence level was high on image (**A**) and moderate on image (**B**)

Slightly more periosteal calcifications were detected on virtual non-contrast images (25/129, 19.4%; 95% confidence interval [CI], 13.3%, 26.8%) than on DECT arthrography images (21/129, 16.3%; 95% CI, 10.7%, 23.4%) but without statistically significant difference (*p* = 0.29). However, diagnostic confidence to detect periosteal calcification was significantly higher on VNC images with median 3 (3–3) compared to DECT-A images with median 3 (3–3) (*p* = 0.009) (Fig. 2). Agreement between reader 1 and 2 was substantial for VNC images (ĸ = 0.77) and excellent for DECT-A images (ĸ = 0.81).

**Fig. 3:**
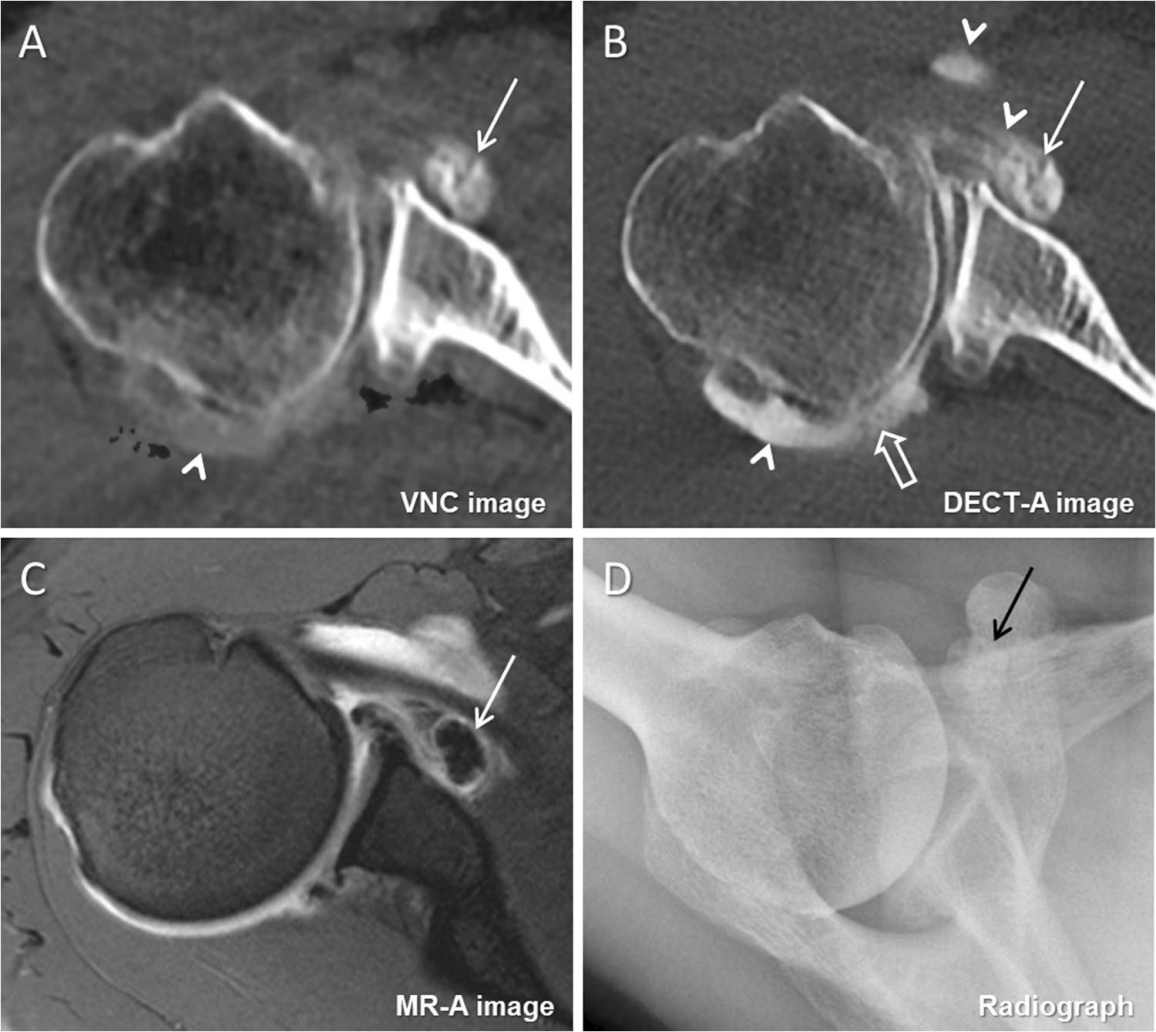
A 37-year-old male with dysplasia of the posterior glenoid and osteoarthritis. On the axial virtual non-contrast image (**A**), a large ossified intraarticular loose body is visible in the subscapular recess of the glenohumeral joint (arrow). On the axial DECT arthrography image (**B**), the intraarticular loose body (arrow) was missed because of similar density as the adjacent iodinated contrast material (arrowheads). The loose body was interpreted as synovitis of the subscapular recess as it is the case for the posterior recess (open arrow). The presence of the intraarticular loose body (arrow) was confirmed on the axial MR arthrography image (**C**) and on the axial radiograph (**D**). Note the incomplete subtraction of iodine (arrowhead) in the posterior recess on image (**A**)

**Fig. 4:**
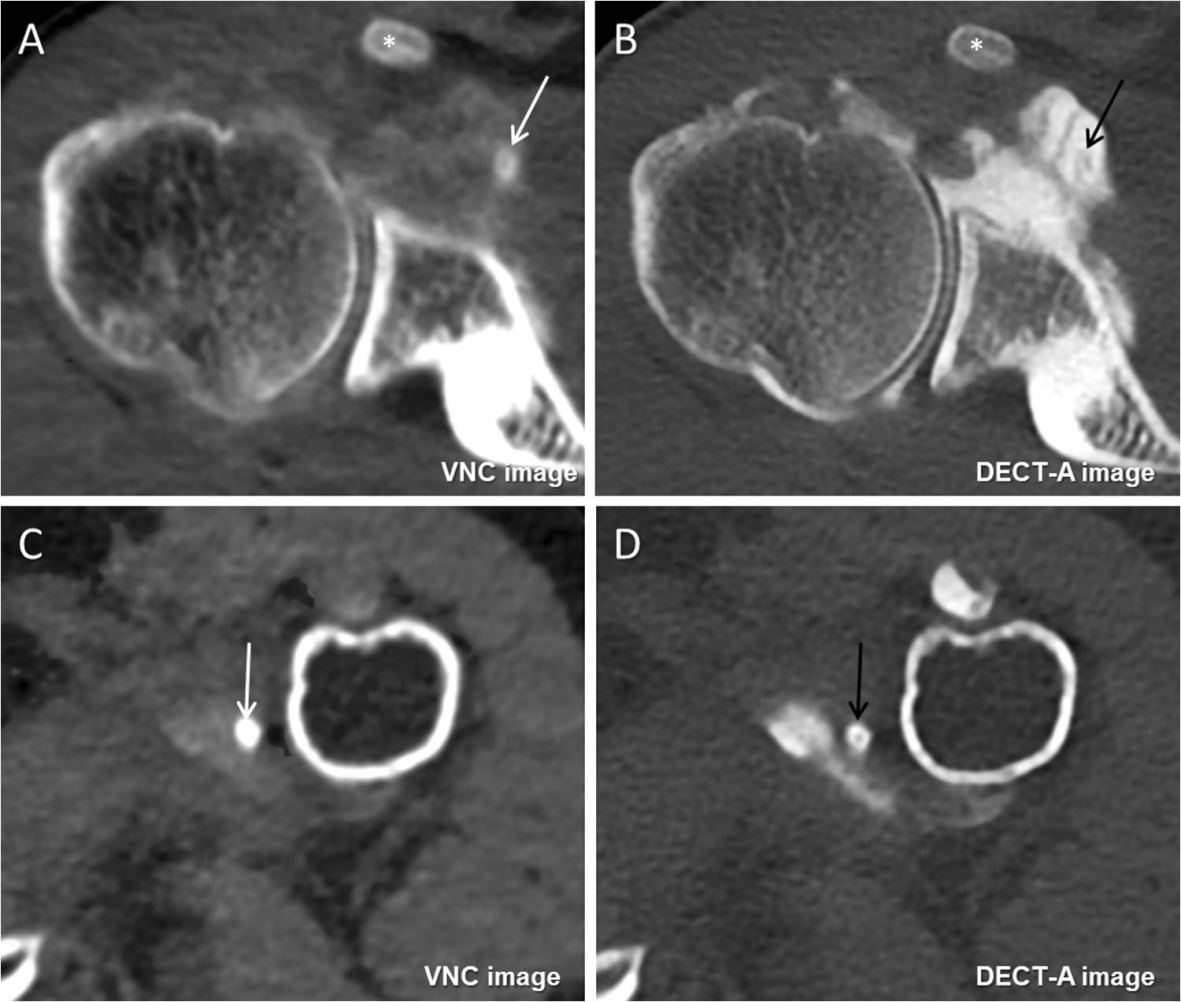
Examples of intraarticular loose bodies on axial virtual non-contrast (**A** and **C**) and DECT arthrography images (**B** and **D**) in two different patients. **A** and **B** A 37-year-old male with recurrent anteroinferior dislocation of the right shoulder. The virtual non-contrast image (**A**) shows a small, moderately calcified intraarticular loose body in the location of the subcoracoid bursa, which was missed on the DECT arthrography image due to a similar appearance as iodine and septations (arrows). **B** and **D** A 57-year-old female with recurrent anteroinferior dislocation of the left shoulder. On the virtual non-contrast image (**C**), a strongly ossified intraarticular loose body in the location of the axillary recess is clearly visible, which was also missed on the DECT arthrography image because of similar density as iodine (arrows). Coracoid process (asterisks in **A** and **B**)

**Fig. 5:**
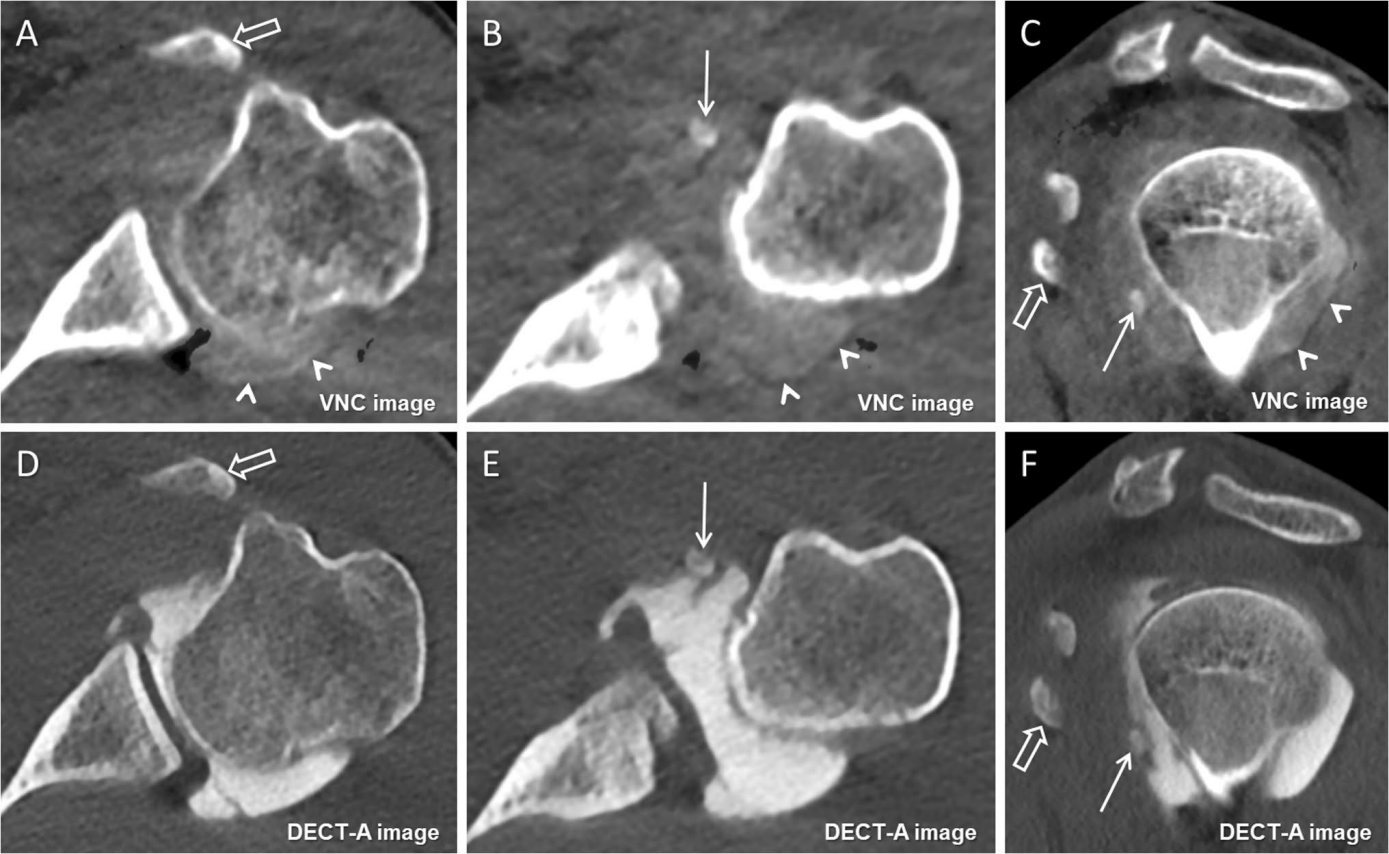
A 31-year-old male with recurrent anteroinferior shoulder dislocation. The axial and sagittal oblique virtual non-contrast image (**A** and **C**) and the axial and sagittal oblique DECT arthrography image (**D** and **F**) both show a displaced fracture fragment from the tip of the coracoid process (open arrow). However, the small ossified intraarticular loose body in the anterior axillary recess (arrow) was missed in the axial and sagittal oblique DECT arthrography images (**E** and **F**) because of indistinguishability from iodinated contrast material but was clearly identified in the axial and sagittal oblique virtual non-contrast images (**B** and **C**). Note the incomplete subtraction of iodine (arrowheads) in the posterior recess on image **A**–**C**

Regarding intraarticular loose bodies, the detection rate was significantly higher on VNC (14/129, 10.9%; 95% CI, 6.4%, 17.1%) than on DECT-A images (7/129, 5.4%; 95% CI, 2.5%, 10.4%) (*p* = 0.02). Diagnostic confidence to detect intraarticular loose bodies was also significantly higher on VNC than on DECT-A images (median 3 (3–4) vs. 3 (3–3)) (*p* < 0.001) (Figs. 3, 4, and 5). Interreader agreement was substantial for DECT-A images (ĸ = 0.73) and excellent for VNC images (ĸ = 0.92).

## Discussion

We showed that virtual non-contrast (VNC) images calculated from DECT shoulder arthrography scans are superior to blended DECT arthrography images for the detection of intraarticular loose bodies and increase the confidence in detecting periosteal calcifications.

In recent years, dual-energy CT applications have increased in musculoskeletal imaging, especially the detection of gout and bone marrow edema, and reduction of metal artifacts has been investigated extensively [9]. In a meta-analysis with 10 included studies evaluating the utility of DECT for diagnosing gout, Gamala et al. reported a pooled sensitivity and specificity of 0.81 and 0.91, respectively [4]. Suh et al. reported a pooled sensitivity and specificity of 0.85 and 0.97, respectively, for bone marrow edema detection with DECT in their meta-analysis including 12 eligible studies [5]. In a study including 31 patients with metal implants, Bamberg et al. showed substantial reduction of metal artifacts with high energy virtual monoenergetic reconstructions from DECT scans compared to the standard image [6]. Up to do date, only a limited number of studies investigated virtual non-contrast images calculated from DECT scans in musculoskeletal imaging [7, 14, 18]. Several non-MSK studies presented successful calculation of virtual non-contrast images from spectral imaging data, which are accurate compared to true unenhanced images. In a study with 25 patients, Laukamp et al. did not find a significant difference in the mean liver attenuation between true unenhanced (54.6 HU ± 10.8 HU), VNC arterial phase (55.7 HU ± 10.8 HU), or VNC venous phase (58.3 HU ± 10 HU) images (*p* > 0.05), acquired with a spectral detector CT. Furthermore, diagnostic assessment of pathology was comparable between datasets [10]. Zhang et al. showed similar results in a study with 102 patients who received liver scans in dual-energy CT technique with no difference in CT numbers (HU) between VNC and true unenhanced images [11]. Chen et al. demonstrated in a study with 171 patients that the diagnostic performance of VNC images in combination with iodine overlay images (single phase, calculated from DECT) was equivalent to combined true unenhanced and blended contrast-enhanced images (dual phase) for the detection of renal masses. Furthermore, the sensitivity to detect renal stones was 87% for VNC images [12]. In a study with a phantom and 21 patients, Si-Mohamed et al. found similar diagnostic performance of VNC und true unenhanced images in detecting aortic intramural hematoma with similar attenuation values (HU) for hematoma and blood [13].

In our study with 123 patients and 129 shoulders, the VNC images were of good quality. The images were calculated in syngo.via from the 80 and 140 kV dataset with the customized liver VNC application, which uses material decomposition for successful iodine removal. With the usage of the virtual unenhanced technique, an intraarticular iodine removal of 91% on average was achieved. However, the density of joint fluid of the shoulder joint is above the density of water (0 HU) on true unenhanced CT, depending on the amount of protein, debris, or even hemorrhage. Therefore, the percentage of intraarticular iodine removal is assumed to be more than the calculated 91%. Sandhu et al. showed the superiority of the virtual unenhanced over the virtual monoenergetic technique for the calculation of VNC images. In their study, they presented a single case of improved visibility of a scapular fracture fragment on VNC images compared to virtual monoenergetic and DECT-A images [14]. Our study results were in accordance with VNC images performing superior to blended DECT arthrography images in the detection of intraarticular loose bodies (14 vs. 7; *p* = 0.02). According to our experience, this applied especially for calcified loose bodies as their density and optical appearance were similar to or indistinguishable from iodinated contrast material. On the other hand, low-density intraarticular loose bodies could still be outlined on VNC images.

Furthermore, not significantly, more periosteal calcifications were detected on virtual non-contrast images, which might be misdiagnosed as labral tears outlined by contrast material on DECT-A images due to a similar appearance. On VNC images, labral tears disappear after iodine subtraction, while periosteal calcifications persist.

For both intraarticular loose bodies and periosteal calcifications, diagnostic confidence was significantly higher on VNC images than on DECT-A images (*p* < 0.001 and *p* = 0.009, respectively).

Besides the ability to calculate VNC images, we were also able to reduce the amount of intraarticular iodinated contrast material for the dual-energy CT scans to 80 mg iodine per milliliter at arthrography without compromise in image quality. The observed image contrast between iodine and soft tissues on DECT-A images was excellent with mean 953 HU, allowing to confidently assess the articular structures (labrum, rotator cuff, cartilage) for pathology. In a study with a phantom and 23 patients, An et al. also observed an image contrast above 800 HU between intraarticular iodine and soft tissues for their optimized virtual monochromatic spectral imaging DECT protocol using 60 mg iodine/ml [19].

The radiation dose of our DECT shoulder arthrography scans with a CTDIvol of mean 12.8 mGy and an effective dose of mean 2.21 mSv were in line with the literature. For their optimized virtual monochromatic spectral imaging DECT protocol in 23 patients, An et al. reported a CTDIvol of 17.8 mGy [19]. In a study with 20 patients receiving 120 kV single-energy non-contrast CT scans of the shoulder, Biswas et al. reported a mean CTDIvol of 19.5 mGy and a mean effective dose of 2.06 mSv [20].

One limitation of our study was that 2 different CT scanners were used for patient scans. However, the same dual-energy protocols were used with adjusted acquisition parameters to equal dose and hence comparable image quality. Furthermore, the scan mode of the 80 kV and the 140 kV CT scan was in sequential technique with a 70-s delay which could not be deactivated in order for the VNC application to work. Patient motion during the 2 CT scans was a potential problem, which indeed we experienced in only one patient with poor VNC image quality. Another limitation was that for the majority of patients, true unenhanced shoulder CT scans were not available for direct comparison with VNC images. Additionally, different reconstruction kernels were used for DECT-A images (bone kernel) and VNC images (soft tissue kernel) which may impact on the visibility of intraarticular loose bodies and periosteal calcifications beyond iodine subtraction. However, we believe that a soft tissue kernel reconstruction for DECT-A images would indeed impair visibility, since the already dense intraarticular iodine would present even brighter resulting in increased indistinguishability. Further, as blinding of image type (VNC vs. DECT-A) during image reading was not possible, comparing the confidence levels might has been subject to bias. Last, the number of detected intraarticular loose bodies on VNC images (14/129) compared to DECT-A images (7/129) in the study group was rather low which may limit generalization of study results. Nevertheless, substantial to excellent interreader agreement underline the findings.

In summary, good quality virtual non-contrast images were calculated form dual-energy CT shoulder arthrography scans with image postprocessing, which are superior to DECT arthrography images for the detection of intraarticular loose bodies and increase the confidence in detecting periosteal calcifications. Radiation dose is comparable to single-energy shoulder CT.

## Supplementary Information

Below is the link to the electronic supplementary material.

Supplementary file1 (DOCX 63 KB)

## Abbreviations

CTDIvol: Volume CT dose index
DECT: Dual-energy CT
DLP: Dose length product
HU: Hounsfield unit
kV: Kilo volt
mAs: Milliampere seconds
mGy: Milligray
mSv: Millisievert
ROI: Region of interest
VNC: Virtual non-contrast
