# Supplementary figures and images for: Virtual non-contrast images calculated from dual-energy CT shoulder arthrography improve the detection of intraarticular loose bodies
Source: Skeletal Radiol. 2022 Feb 11;51(8):1639–47. doi: 10.1007/s00256-022-04007-7 (PMC9197803; doi:10.1007/s00256-022-04007-7)

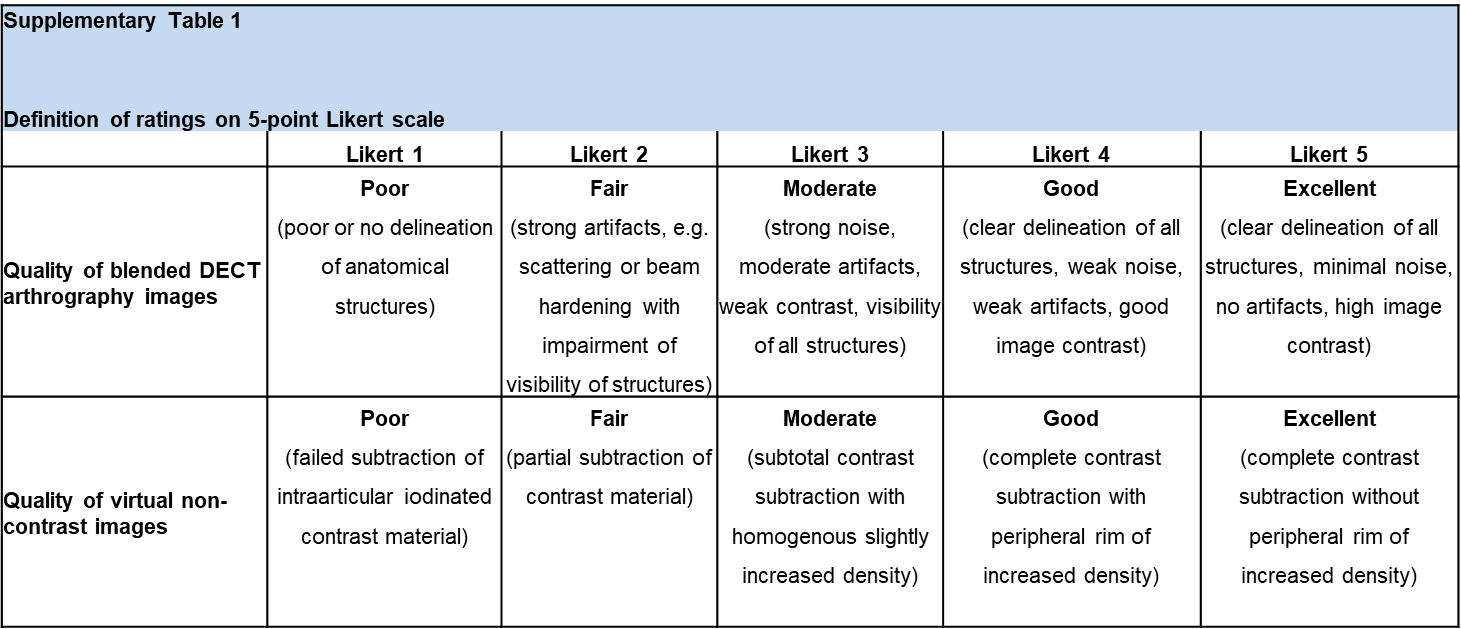

Supplement: Supplementary file 1 — Supplementary file1 (DOCX 63 KB) [file 256_2022_4007_MOESM1_ESM.docx]
